# Approach to Selective Dry Cow Therapy in Early Adopter Italian Dairy Farms: Why Compliance Is So Important

**DOI:** 10.3390/ani13223485

**Published:** 2023-11-11

**Authors:** Marcello Guadagnini, Clarissa Gogna, Cecilia Tolasi, Giacomo Tolasi, Gisella Gnali, Gustavo Freu, Anoar Jamai Masroure, Paolo Moroni

**Affiliations:** 1 Axiota Animal Health, 2809 East Harmony Road #190, Fort Collins, CO 80528, USA; marcello.guadagnini@multimin.eu; 2 Department of Veterinary Medicine and Animal Sciences—DIVAS, University of Milan, 26900 Lodi, Italy; gognaclarissa@gmail.com (C.G.); gustavofreu@usp.br (G.F.); anoar.jamai@unimi.it (A.J.M.); 3 Studio Veterinario “Armigio” Scarpizzolo di San Paolo, 25020 Brescia, Italy; tolasicecilia@gmail.com (C.T.); giacomotolasi@gmail.com (G.T.); gise.gnali@gmail.com (G.G.); 4 Department of Animal Nutrition and Production, School of Veterinary Medicine and Animal Science, University of Sao Paulo (USP), Pirassununga 13635-900, SP, Brazil; 5 Quality Milk Production Services, Animal Health Diagnostic Center, Cornell University, Ithaca, NY 14853, USA

**Keywords:** selective dry cow therapy, compliance, Italian dairy farms

## Abstract

**Simple Summary:**

Selective dry cow therapy (SDCT) involves selecting only cows or mammary quarters with existing intramammary infection to be treated with antimicrobials at dry-off. SDCT became mandatory in Italy in January 2022. Previously, the most common practice was to treat all cows at dry-off with intramammary antibiotics to address current udder infections and to prevent new ones. This study describes the challenges of 11 herds with SDCT, focusing on owner compliance. Compliance, the ability of the farmer to follow veterinary advice, was a critical issue as 21% of all cows were non-compliant, highlighting the need for better treatment monitoring. At first testing, non-compliant cows were 3.77 times more likely to have subclinical mastitis compared to compliant cows. Observations of veterinarians and farmers showed a lack of monitoring systems for cows to be treated with antibiotic selections. This study suggests the need for improved education and increased veterinarian involvement in the implementation of SDCT.

**Abstract:**

Selective dry-cow therapy (SDCT) became mandatory in Italy on 28 January 2022. During 2020, a group of farms involved in a milk quality program began a pilot experiment with SDCT in order to understand its challenges and to identify areas for procedural improvements. The aim of this study was to describe the challenges and results of the SDCT in early adopters’ herds with a special focus on treatment compliance. Retrospective data from 1911 cows from 11 dairy herds were evaluated. Somatic cell counts, clinical mastitis (CM) history, and the California Mastitis Test (CMT) were used as criteria for SDCT. Based on the dairy herd improvement test results and CM history, 48% of all cows should have received antibiotic treatments and internal teat sealants. Adding the CMT at dry-off increased the percentage of antibiotic-treated cows to 62%, with relevant variation among farms. Concerning treatment compliance, 21% of the cows were “non-compliant”, suggesting the importance of monitoring treatment compliance. In conclusion, even if commonly used selection criteria for antibiotic treatments were used, the need for more education and in-depth monitoring of the SDCT adoption process was clearly identified. Close collaboration and agreement between veterinarians and farmers are key for SDCT adoption success.

## 1. Introduction

Increased frequency of antimicrobial resistance affects both human and veterinary medicine and should not be underestimated in food-producing animals [1]. It is suggested that selective dry-cow therapy (SDCT) in the dairy sector is one of the measures that should be implemented for a more controlled usage of antibiotics [2,3]. With SDCT, only cows or quarters with existing intramammary infection (IMI) are selected for treatment with intramammary antimicrobials during dry-off [4,5]. This procedure is compulsory in Italy and is in agreement with European Guidelines for the prudent use of antimicrobials in veterinary medicine (2015/C 299/04 [6] and the Italian National Plan to reduce Antimicrobial Resistance 2017–2020 [7]). Identifying cows to be treated with antibiotics at dry-off is crucial for farmers, practitioners, and health authorities. There has been limited research exploring what Italian farmers know and think about SDCT. Research in other countries has demonstrated that SDCT can be accomplished without negative consequences on udder health [3,8,9]. It may be beneficial to understand how Italian farmers apply SDCT, how it affects udder health, and the hurdles during the adoption process. Besides the selection criteria for antibiotic treatments, one of the key aspects is how this selection is applied and how veterinary recommendations are followed.

Compliance is the willingness to follow a prescribed course of treatment. Compliance depends upon farmers understanding and following recommendations made by veterinarians and team members for diagnostic tests, treatment, and preventive health care. In other words, compliance is the farmer’s ability to properly follow veterinary advice on dry-cow treatments. The World Health Organization estimates that only 50% of human patients comply with treatment recommendations [10]. The concept of compliance is not widely explored in bovine health practices as its meaning is somewhat ambiguous. The definition of a consistent and approved procedure to identify cows to be treated at dry-off is crucial for farmers who need to reduce IMI after calving and health authorities who must verify that farms are complying with antimicrobial use regulations on dairy farms. A procedure to identify cows to be treated at dry-off should meet several criteria. It should be sufficiently accurate, easy to perform and interpret, inexpensive, and relatively safe and should have customized thresholds based on the udder health status of each specific herd.

The aim of this study was to describe SDCT outcomes on udder health and adoption challenges on 11 early adopter herds. This study places special emphasis on dry-cow treatment compliance and how it affects the udder health status in the subsequent lactation.

## 2. Material and Methods

For this study, data from 11 dairy herds already involved in a milk quality service program with the Armigio Veterinary Group were collected. Those herds began a pilot experiment with SDCT before it became mandatory in order to understand the challenges and contribute to the improvement of this procedure. Dairy herds were located in Northern Italy, and all herds were using dairy herd improvement testing (DHI) and applying SDCT protocols for all of 2020 under veterinary supervision. Herds had a history of testing negative for *Staphylococcus aureus* and *Streptococcus agalactiae*. Detailed herd characteristics are shown in Table 1. Milk production average was 36 kg/cow/day (varying from 32 to 42 kg/cow/day). Only 3 herds implemented diet changes aimed at reducing milk production prior to dry-off. No vaccination programs against mastitis pathogens were in place on any farm, and internal teat sealant (ITS) was used in all cows on every farm.

A total of 1911 multiparous cows (43% second lactation, 57% third and greater lactation) were included in the study and each lactation record began with a calving date between 1 January 2020 and 31 December 2020. The DHI data were provided by the National Breeders Association (AIA) in a text format and absorbed into Dairy Comp 305^®^ (Valley Agricultural Software, Tulare, CA, USA). DHI tests were performed from 8 to 11 times a year with intervals that could range from 33 to 45 days (Table 1). Lactation records for each farm included cow and farm ID, milking type and frequency, parity, calving date, breed, dry period length, and culling dates. The data on milk production during lactation, and somatic cell count (SCC) from at last 3 tests before dry-off and from the first test of the following lactation were also available. Disease information was not available from this source.

Additional information regarding dry-off treatment and clinical mastitis (CM) history during the previous lactation was collected by farmers and vets using a specific Excel^®^ (Microsoft Office 365, Microsoft Corp., Redmond, WA, USA) spreadsheet. The final dataset was obtained by joining DHI data with the ones collected on farms. For the analysis, SCC data were log transformed into linear score (LS) according to [11].

Based on Buiatric Veterinary Scientific Association (SIVAR) guidelines [12] for SDCT and Armigio Veterinary Group personal experiences, the indication for an antibiotic treatment and ITS at dry-off was for cows that had at least one of last three tests before dry-off above 100,000 SCC/mL for parity = 1 and 200,000 SCC/mL for parity >1 and a history of CM or a positive California Mastitis Test (CMT) at dry-off. CMT was performed at dry-off for cows that were meant to receive ITS based on the LS and CM information in order to check the SCC content of each quarter. All cows with none of the last 3 DHI tests above the beforementioned SCC criteria, no CM, and a negative CMT at dry-off should have been treated with ITS only.

The percentage of cows that met the criteria for antibiotic treatment based on SCC at the last 3 DHI tests and presence of CM, and the percentage of actual antibiotic treatments based on SCC, CM, and CMT results were estimated. In order to address SDCT outcome, the percentage of subclinical mastitis (SCM) at first DHI test and udder health indicators (healthy, new infection, chronic, and cured cows) were calculated according to [13].

To evaluate treatment compliance, we calculated the proportion of cows that should have received antibiotic treatment and ITS according to the established criteria but only received ITS. Cows that were correctly treated with antibiotic and ITS were defined as “compliant”, while cows that should have been treated with antibiotic but only received ITS were defined as “non-compliant”. We chose to evaluate this portion of compliance because cows in the dataset that were treated with antibiotics were meeting the selection criteria based on SCC history and/or CM or were positive for CMT.

Udder health risk of non-compliance was evaluated. For this, a logistic regression model was created to assess the SCM risk at the first DHI test for non-compliant cows compared to compliant ones. Cows were considered to have SCM when presenting >200,000 SCC/mL (LS at first DHI test >4) [14]. SCM (yes/no) was included in the model as an independent variable. Fixed dependent variables were SCC at last 3 tests, parity, calving month, previous 305 days milk mature equivalent, dry period length, days in milk at first DHI test, previous lactation SCM (yes vs. no), and treatment compliance (compliant vs. non-compliant). Herd was included as a random effect in the model. Odds ratios for SCM were calculated. Analyses were performed with JMP 15^®^ statistical analysis software (SAS Institute Inc., Cary, NC, USA), and *p*-values < 0.05 were considered significant.

At the end of the experiment, a summary questionnaire was completed by both vets and farmers to gain more insights into the SDCT procedure adoption.

## 3. Results and Discussion

Considering DHI tests and CM data only, 48% (*n* = 923/1911) of the cows should have received the antibiotics and ITS and 52% (*n* = 988/1911) of the cows should have received ITS only. Thirteen cows had missing information on their dry-off treatment due to involuntary culling before the event and do not appear in the treatment figures (Table 2). Adding CMT at dry-off increased the percentage of antibiotic-treated cows to 62%, with a relevant variation among herds. The combination of these two sources of information could partially explain the increased proportion of cows treated with antibiotics.

On average, the last DHI test occurred 23 ± 18 days before dry-off, with 25% of cows having an interval between the last test and dry-off greater than 31 days. This relatively large timeframe might explain why CMT detected a higher proportion of cows with SCM, compared to DHI tests. Despite CMT having relatively low sensitivity [15], as the test is performed at the quarter level, it might be able to overcome the dilution effect of the DHI composite sample. Although there was an increase in antibiotic use due to the CMT addition to SCC and CM, there was a noticeable decrease (38%) in antibiotic treatments compared to 100% of the blanket dry cow treatment.

Cows with SCM at first DHI testing made up 22% overall, ranging from 13% to 31% at the single farm level (Table 3). Cows culled before the first DHI test or having missing data made up 10% (*n* = 190/1911). Considering udder health dynamics by comparing the last test before dry-off and the first test after calving, the new infection rate was 16% and healthy cows made up 64% (Table 3). There were some herds (e.g., herd 5 and 10) who had new infection rates, >20%. These results suggest that there is room for improvement in dry-off procedures and in dry and fresh cow management in order to lower IMI pressure.

Out of the 923 cows included in the compliance assessment, 21% were “non-compliant” (Table 4). At farm level, the lowest “non-compliance” rate was 3.9% while the highest was 69%. It remains unclear if the decision to not treat a cow with antibiotics is just a mistake in the routine or a conscious evaluation.

Cows that failed to be treated with antibiotics are especially important because failing to treat cows that are meeting any SDCT criteria might pose a greater risk for udder health. Non-compliant cows were 3.77 times (95% C.I. 2.18–6.54) more likely to have SCM at first DHI test compared to compliant cows.

The questionnaire results showed that SDCT procedures were explained verbally in 10 of the 11 herds and in a training format in one herd. After relaying to farmers the results of the study, both vets and farmers revealed in the survey that they were unaware of the compliance deviation until data analysis was performed and the results were shared with them. In 91% of cases (10 out of 11 herds), the compliance deviation was attributed to a lack of a monitoring system.

From this study, we were able to obtain more insights into how SDCT is adopted on early adopters’ Italian farms. A close collaboration between veterinarians and farmers is needed. Veterinarian tasks include carefully assessing and continuously evaluating the herd udder health status, while communicating and mutually agreeing with the farmer on the procedures to be used. The practical application of a vet’s advice is managed by the farmer, who must face the complexity of adopting different procedures based on cow’s health. Much data are available to assess the health status of the cows, minimizing discretion.

Overall, the percentage of SCM at first DHI test and new infections often exceeded commonly used goals, especially on some farms [16,17]. As implementing SDCT implies having several cows going through the dry period without the protection of antibiotic treatment, there is a greater need for improved housing and management conditions for dry and fresh cows. This will minimize infection pressure, and cow immunity will be enhanced. The use of ITS without antibiotics may require more careful hygiene during administration at dry-off.

Several studies have demonstrated the validity of cows’ selection for antibiotic treatment based on algorithms [8,18]. Data quality deriving from DHI testing frequency, CM detection, and good data recording are key for making correct decisions. The amount of data involved in this process is difficult to manage using paper records so computer software might ease data handling and reduce human errors.

Based on the complexity of the task, establishing treatment criteria without an adequate follow-up will not guarantee the success of SDCT. Constant monitoring and treatment compliance assessments are needed.

Without a high level of compliance, it may be difficult to critically evaluate if treatment selection criteria are adequate. As with many other milk quality tasks, SDCT should be the subject of routine training sessions for the farmer and employees who often execute dry-off treatment and add subjectivity to the selection process.

Within the communication and training field, it might also be useful, especially where many employees are present, to have written, easy-to-read procedures and to clearly define how the assigned treatment list is communicated to those responsible for the task.

## 4. Conclusions

SDCT on Italian dairy farms has been implemented according to legislation, but there is room for improvement on the udder health profile, especially on some of the herds involved. Twenty-one percent of the cows were “non-compliant”, suggesting the importance of monitoring treatment compliance, as this can negatively impact udder health. A large proportion of veterinarians and farmers attributed the compliance deviation to the lack of a monitoring system. Close collaboration and alignment between veterinarians and farmers are key for SDCT adoption success.

## Figures and Tables

**Table 1:** Descriptive characteristics of 11 dairy farms applying SDCT involved in the study.

Farm	Lactating Cows (*n*)	Milking Parlor ^1^	DHI SCC ^2^ (Average)	DPL ^3^	DHI ^4^	Milk Production Average (Liters/305 d)
1	419	Conventional	257,000	66	11	11,441
2	112	Conventional	265,000	56	8	10,172
3	297	Conventional	241,000	65	8	12,190
4	165	Conventional	308,000	65	11	9751
5	343	Conventional	271,000	67	9	10,002
6	253	Conventional	198,000	57	10	11,443
7	133	Conventional	354,000	58	11	10,822
8	198	AMS	263,000	44	8	10,755
9	119	Conventional	169,000	63	8	10,312
10	251	Conventional	276,000	59	8	11,298
11	269	AMS	261,000	61	8	11,940
Average	233	-	260,273	60	9	10,918

^1^ AMS: automatic milking system. ^2^ Somatic cell count from dairy herd improvement testing. This average includes milk produced by cows with mastitis under treatment. ^3^ Dry period length. ^4^ Dairy herd improvement testing frequency per year.

**Table 2:** Dry-off treatment percentages by farm assigned based on SCC and CM data only (left columns) and dry-off treatments assigned based on SCC, CM, and CMT (right columns). The treatments on the right columns are the actual ones.

Farm	Treatment Assignment Based on DHI ^1^ and CM ^2^ Data		Actual Treatments Based on DHI Data + CM + CMT ^3^	
	AB + ITS ^4^ % (*n*)	ITS ^5^ % (*n*)	AB + ITS % (*n*)	ITS % (*n*)
1	49 (150)	51 (157)	64 (193)	36 (108)
2	30 (26)	70 (60)	69 (59)	31 (26)
3	52 (139)	48 (129)	73 (196)	27 (72)
4	44 (59)	56 (76)	71 (96)	29 (39)
5	36 (86)	64 (150)	65 (154)	35 (82)
6	45 (85)	55 (104)	59 (110)	41 (77)
7	67 (63)	33 (31)	68 (64)	32 (30)
8	60 (87)	40 (59)	22 (31)	78 (111)
9	44 (32)	56 (41)	63 (46)	37 (27)
10	38 (55)	62 (89)	46 (66)	54 (78)
11	61 (141)	39 (92)	73 (171)	27 (62)
All	48% (923)	52% (988)	62% (1186)	38% (712)

^1^ Dairy herd improvement testing. ^2^ Clinical mastitis. ^3^ California Mastitis Test. ^4^ Antibiotic treatment and internal teat sealant. ^5^ Only internal teat sealant administration.

**Table 3:** Percentage of SCM at first DHI test and udder health indicators of 11 dairy farms using SDCT.

Farm	SCM ^1^ at First DHI ^2^ Test (Yes/No)		SCC Dynamics: ^3^ Last Test before Dry-Off vs. First Test after Calving			
	Yes, % (*n*)	No, % (*n*)	Healthy (%)	New Infection (%)	Chronic (%)	Cured (%)
1	22 (62)	78 (225)	60	14	8	18
2	14 (11)	86 (67)	80	11	1	8
3	23 (58)	77 (193)	63	16	7	14
4	22 (27)	78 (98)	65	16	6	13
5	31 (56)	69 (126)	61	28	5	6
6	20 (36)	80 (142)	69	15	6	10
7	20 (18)	80 (71)	59	11	9	21
8	16 (21)	84 (113)	65	11	5	19
9	13 (9)	87 (58)	70	6	8	16
10	28 (38)	72 (98)	63	24	4	9
11	23 (44)	77 (150)	56	15	7	22
All	22 (380)	78 (1341)	64	16	6	14

^1^ Subclinical mastitis. ^2^ Dairy herd improvement testing. ^3^ Udder health indicators (healthy, new infection, chronic, and cured cows) were analyzed according to [13].

**Table 4:** Percentage of compliant and non-compliant cows by farm.

Farm	Compliant ^1^, % (*n*)	Non-Compliant ^2^, % (*n*)
1	89 (133)	11 (17)
2	96 (25)	4 (1)
3	92 (128)	8 (11)
4	92 (54)	8 (5)
5	77 (66)	23 (20)
6	81 (69)	19 (16)
7	78 (49)	22 (14)
8	31 (27)	69 (60)
9	87 (28)	13 (4)
10	56 (31)	44 (24)
11	85 (120)	15 (21)
Total	79 (730)	21 (193)

^1^ Cows that were correctly treated with antibiotic and ITS. ^2^ Cows that were meant to be treated with antibiotic and ITS but received only ITS.

## Data Availability

The raw data of this study will be made available by the authors (corresponding author) to any qualified researcher.

## References

[1] Nobrega D.B., Naqvi S.A., Dufour S., Deardon R., Kastelic J.P., De Buck J., Barkema H.W. (2020). Critically Important antimicrobials are generally not needed to treat nonsevere clinical mastitis in lactating dairy cows: Results from a network meta-analysis. J. Dairy Sci..

[2] Rajala-Schultz P., Nødtvedt A., Halasa T., Persson Waller K. (2021). Prudent use of antibiotics in dairy cows: The nordic approach to udder health. Front. Vet. Sci..

[3] Rowe S., Kabera F., Dufour S., Godden S., Roy J.P., Nydam D. (2023). Selective dry-cow therapy can be implemented successfully in cows of all milk production levels. J. Dairy Sci..

[4] Cameron M., Keefe G.P., Roy J.-P., Stryhn H., Dohoo I.R., McKenna S.L. (2015). Evaluation of selective dry cow treatment following on-farm culture: Milk yield and somatic cell count in the subsequent lactation. J. Dairy Sci..

[5] Scherpenzeel C.G.M., den Uijl I.E.M., van Schaik G., Riekerink R.G.M.O., Hogeveen H., Lam T.J.G.M. (2016). Effect of different scenarios for selective dry-cow therapy on udder health, antimicrobial usage, and economics. J. Dairy Sci..

[6] EU Guidelines for the Prudent Use of Antimicrobials in Veterinary Medicine. *Off. J. Eur. Union* 2015, C 299/7. https://health.ec.europa.eu/system/files/2016-11/2015_prudent_use_guidelines_en_0.pdf.

[7] (2017). National Action Plan on Antimicrobial Resistance (PNCAR) 2017–2020. https://www.salute.gov.it/imgs/C_17_opuscoliPoster_362_ulterioriallegati_ulterioreallegato_0_alleg.pdf.

[8] McCubbin K.D., de Jong E., Lam T.J.G.M., Kelton D.F., Middleton J.R., McDougall S., De Vliegher S., Godden S., Rajala-Schultz P.J., Rowe S. (2022). Invited review: Selective use of antimicrobials in dairy cattle at drying-off. J. Dairy Sci..

[9] Tijs S.H.W., Holstege M.M.C., Scherpenzeel C.G.M., Santman-Berends I.M.G.A., Velthuis A.G.J., Lam T.J.G.M. (2022). Effect of selective dry cow treatment on udder health and antimicrobial usage on dutch dairy farms. J. Dairy Sci..

[10] Yach D. (2003). Adherence to Long-Term Therapies.

[11] Schukken Y.H., Wilson D.J., Welcome F., Garrison-Tikofsky L., Gonzalez R.N. (2003). Monitoring udder health and milk quality using somatic cell counts. Vet. Res..

[12] (2019). Il Trattamento Dell’asciutta: Linea Guida Essenziale.

[13] Fauteux V., Roy J.P., Scholl D.T., Bouchard É. (2014). Benchmarks for evaluation and comparison of udder health status using monthly individual somatic cell count. Can. Vet. J..

[14] Dohoo I.R., Leslie K.E. (1991). Evaluation of changes in somatic cell counts as indicators of new intramammary infections. Prev. Vet. Med..

[15] Dingwell R.T., Leslie K.E., Schukken Y.H., Sargeant J.M., Timms L.L. (2003). Evaluation of the california mastitis test to detect an intramammary infection with a major pathogen in early lactation dairy cows. Can. Vet. J..

[16] Reneau J.K. (1986). Effective use of dairy herd improvement somatic cell counts in mastitis control. J. Dairy Sci..

[17] Ruegg P.L., Pantoja J.C.F. (2013). Understanding and using somatic cell counts to improve milk quality. Irish J. Agric. Food Res..

[18] Rowe S.M., Nydam D.V., Godden S.M., Gorden P.J., Lago A., Vasquez A.K., Royster E., Timmerman J., Thomas M.J., Lynch R.A. (2021). Partial budget analysis of culture- and algorithm-guided selective dry cow therapy. J. Dairy Sci..

